# Antiseptic Inunction in Scarlet Fever

**Published:** 1893-07-08

**Authors:** J. Brendon Curgenven

**Affiliations:** Teddington Hall


### EDITOR'S LETTER-BOX.

[Our correspondents are reminded that prolixity is a great bar to publication, and that brevity of style and conciseness of statement greatly facilitate early insertion.]

### ANTISEPTIC INUNCTION IN SCARLET FEVER.

Sir,—The last paragraph in Dr. Bertram Roger's letter explains the reason of his failure in treating his cases of scarlet fever. That which he used for inunction was the oleusaban "B," which, as I stated in my last letter, is only used for disinfecting and deoderising closets, commodes, &c., and he could not have noticed the label which states this. The "A" quality is that which is used for inunction, it entirely volatilises, and so acts as an aerial disinfectant. The "B" is an emulsion, and as such is useful for the purposes named, but while it would disinfect the skin it would not give off enough vapour to disinfect the breath.—Yours truly,

J. Brendon Curgenven.

Teddington Hall, July 2nd.

